# Human methionine synthase A2756G polymorphism increases susceptibility to prostate cancer

**DOI:** 10.18632/aging.101509

**Published:** 2018-07-31

**Authors:** Hong-Bao Shao, Kewei Ren, Sheng-Lin Gao, Jian-Gang Zou, Yuan-Yuan Mi, Li-Feng Zhang, Li Zuo, Atsushi Okada, Takahiro Yasui

**Affiliations:** 1 Department of Urology, Third Affiliated Hospital of Nantong University, Wuxi 214041, China; 2 Department of Orthopedics, the Affiliated Jiangyin Hospital of Southeast University Medical School, Jiangyin 214400, China; 3 Department of Urology, The Affiliated Changzhou No. 2 People's Hospital of Nanjing Medical University, Changzhou 213003, Jiangsu Province, China; 4 Department of Nephrourology, Nagoya City University Graduate School of Medical Sciences, Aichi 4678601, Japan; * Equal contribution

**Keywords:** methionine synthase, genetic variation, prostate cancer, polymorphism, ELISA

## Abstract

Background/Aims: Previous results on the association between MTR gene A2756G polymorphism and PCa risk are inconclusive.

Methods: We used odds ratios (ORs) with corresponding 95% confidence intervals (95% CIs) to evaluate the correlation between MTR A2756G polymorphism and risk of PCa in meta-analysis. Serum expression of MTR was detected by ELISA and *in-silico* tools were utilized to assess this variant.

Results: Our study included 2,921 PCa patients and 3,095 control subjects. The results indicated that the MTR A2756G polymorphism is linked with an increased risk of PCa using three genetic models (G-allele vs. A-allele: OR = 1.16, 95%CI = 1.04 - 1.30; GA vs. AA: OR = 1.17, 95%CI = 1.02 - 1.33; GG+GA vs. AA: OR = 1.18, 95%CI = 1.04 - 1.34). Stratified analysis produced similar results. A significant association was also indicated in advanced PCa from the meta-analysis. Finally, our experiments showed evidence that serum MTR levels in PCa patients with AA genotypes were statistically higher than in those with GG/GA genotypes.

Conclusions: Our present study suggests that the MTR A2756G polymorphism may contribute to the risk of developing PCa, particularly in Asian and hospital-based studies. Moreover, serum MTR might be utilized in diagnosis of PCa.

## Introduction

Prostate cancer (PCa) remains the most common carcinoma in males and the sixth leading cause of tumor-associated deaths in Western countries [1]. Although the occurrence of PCa in Asia is much lower than that in developed countries, the incidence and mortality rate of the cancer has been increasing remarkably probably due toimproved diet, westernized lifestyles and development of screening technique [2–4]. To date, numerous factors including family history, prostate specific antigen (PSA) test, and age have been identified as strong predictors for PCa susceptibility; nevertheless, the underlying etiology remains unclear [5,6]. Recently, more and more research has indicated that genetic background, such as genetic variants, may play a vital role in prostate carcinogenesis [7,8].

Previous studies have indicated that folate-metabolizing genes play a significant role in carcinogenesis through DNA methylation [9–11]. Aberrant DNA methylation has been thought to be associated with PCa initiation by inactivating tumor suppressor genes (such as P53) or stimulating proto-oncogenes (such as Ras) [12,13]. Human methionine synthase (MTR), located on chromosome 1 (1q43), is one of the core factors in the folate pathway [14,15]. The MTR gene encodes a protein containing 1265 amino acids (140.5 kDa), which acts as a key enzyme in DNA repair, synthesis, and methylation [16]. Published epidemiological studies have been performed to test whether the MTR A2756G (rs1805087) variant is associated with PCa risk; however, the results remain inconsistent rather than conclusive [17,18]. There were several inconsistent results on the association between MTR A2756G polymorphism and PCa from various case-control studies. Therefore, we performed an updated meta-analysis with accumulated data from all eligible researches to enhance the statistical powers of previous studies and ascertain precise correlation between the MTR variant and PCa risk [18–24]. Furthermore, we enrolled 200 newly diagnosed PCa patients in our centers and investigated the serum expression of MTR by ELISA and utilized in-silico tools to confirm the results of our meta-analysis.

## RESULTS

### Study characteristics

**Figure 1:**
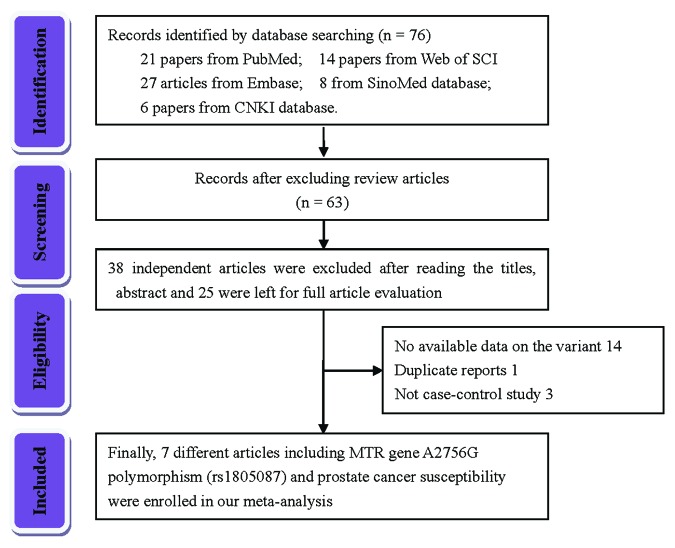
Flow chart of the strategy of literature search among the related studies.

**Figure 2:**
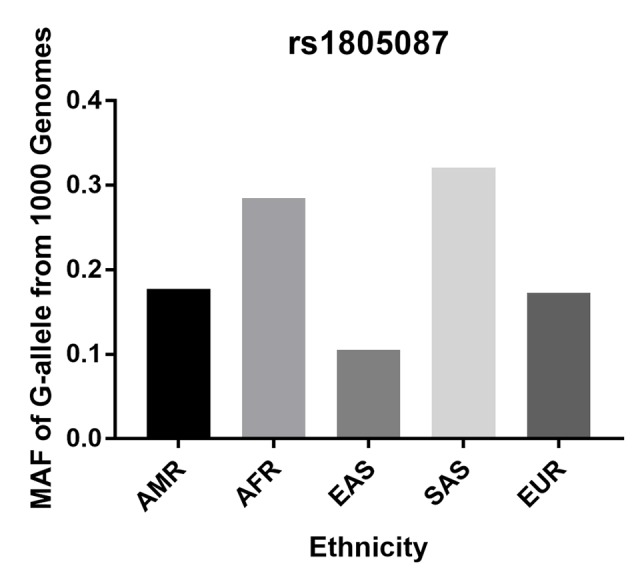
**Minor allele frequencies (MAF) for MTR rs1805087 A2756G polymorphism in control stratified by ethnicity.** Vertical line, minor allele frequency of G-allele from 1,000 Genomes; Horizontal line, ethnicity type; American (AMR), African (AFR), East Asian (EAS), South Asian (SAS), and European (EUR).

Initially, 76 articles were retrieved according to the selection strategy (Fig. 1). Among them, 13 articles were excluded because they were review articles. Then, 38 articles were removed after screening of titles and abstracts. Another 14 articles were excluded because they did not provide available data on the MTR A2756G variant. Among the remaining 11 articles, one article was a duplicate report and 3 articles contained no information about a control group. As a result, a total of 7 articles containing 2,921 PCa patients and 3,095 control subjects concerning the MTR A2756G polymorphism were enrolled in our meta-analysis. Relevant parameters for the selected studies are summarized in Table 1. For the selected seven studies, three were performed on a Caucasian population, three were carried out on an Asian population and only one study was performed concerning South-Americans. In stratified analysis by source of control, HB controls were investigated in five of these studies and the rest were performed on PB controls. For the genotyping methods, three studies used the classical RFLP while the rest utilized real-time PCR and TaqMan. Furthermore, we checked the Minor Allele Frequency (MAF) of MTR A2756G reported for the main worldwide populations. For American (AMR) population: A=0.823, G=0.177; for African (AFR) population: A=0.716, G=0.284; for East Asian (EAS) population: A=0.895, G=0.105; for South Asian (SAS) population: A=0.679, G=0.321; and European (EUR) population: A=0.827, G=0.173 (Fig. 2).

**Table 1:** Study characteristics of MTR rs1805087 A2756G polymorphism included in this meta-analysis.

First author	Year	Country	Ethnicity	Source	Genotyping	Sample size of case					Sample size of control					Average age		*P*_HWE_	NOS
					methods	GG	GA	AA	MAF	Total	GG	GA	AA	MAF	Total	Case	Control		
Ebrahimi	2017	Iran	Asian	HB	PCR-RFLP	13	53	34	0.395	100	6	37	57	0.245	100	NA	NA	0.999	7
Qu	2016	China	Asian	HB	real-time PCR	20	316	1481	0.098	1817	15	319	1692	0.086	2026	66.7 ± 7.2	66.9 ± 6.8	0.993	9
López-Cortés	2013	Ecuador	SA	PB	PCR-RFLP	3	9	92	0.072	104	1	4	105	0.027	110	NA	NA	0.001	9
Weiner	2012	Russia	Caucasian	PB	real-time PCR	15	134	221	0.222	370	16	96	173	0.225	285	69 ± 8.0	59 ± 17	0.580	7
Cai	2010	China	Asian	HB	PCR-RFLP	5	27	185	0.085	217	3	29	188	0.080	220	72.36 ± 12.16	72.83± 12.27	0.139	7
Marchal	2008	Spain	Caucasian	HB	Taqman	9	54	118	0.199	181	11	55	138	0.189	204	70.7 ± 7.29	70.3 ± 7.82	0.088	7
Kimura	2000	Germany	Caucasian	HB	PCR-RFLP	4	41	87	0.186	132	4	44	102	0.173	150	65.6 ± 6.0	62.0 ± 11.4	0.773	9

SA: South America; HWE: Hardy-Weinberg equilibrium of controls, HB: Hospital-based; PB: Population-based; MAF: Minor allele frequency; NOS: Newcastle-Ottawa Scale; RFLP: restriction fragment length polymorphism; NA: not available.

### Meta-analysis

**Figure 3:**
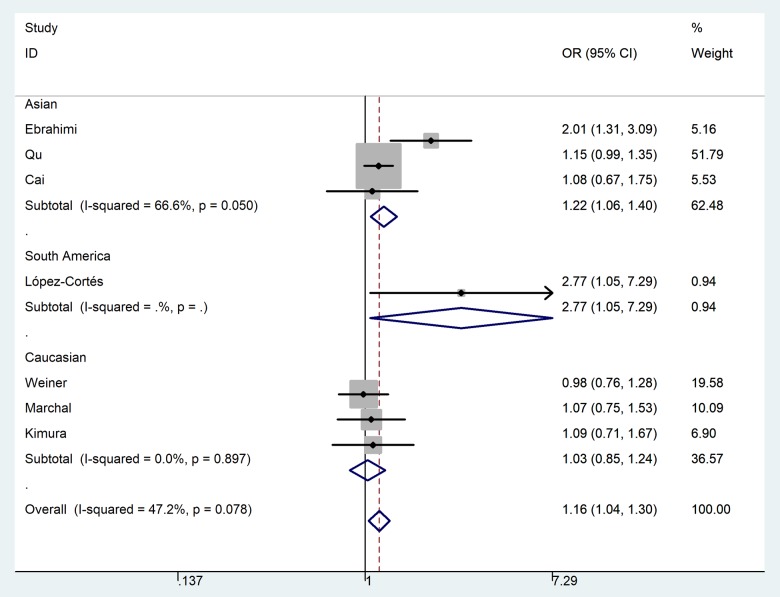
**Forest plot of PCa risk associated with the MTR A2756G polymorphism (allelic contrast of G-allele vs. A-allele, fixed-effects) in the stratified analyses by ethnicity**. The *squares* and *horizontal lines* represent the study-specific OR and 95% CI. The area of the *squares* reflects the weight (inverse of the variance). The *diamond* corresponds to the summary OR and 95% CI. Separate details were summarized in Table 2.

**Figure 4:**
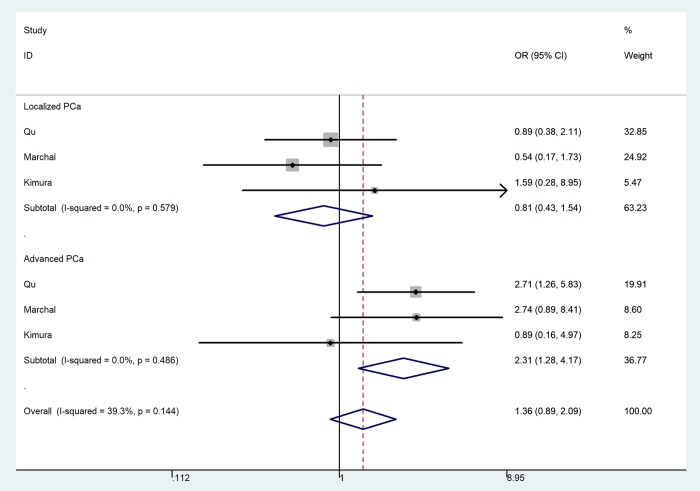
**Forest plot of prostate cancer risk associated with the MTR A2756G polymorphism (recessive model of GG vs. GA+AA, fixed-effects) in the stratified analyses by tumor stage.** The *squares* and *horizontal lines* represent the study-specific OR and 95% CI. The area of the *squares* reflects the weight (inverse of the variance). The *diamond* corresponds to the summary OR and 95% CI.

In the pooled analysis of all selected studies, an increased risk of PCa was associated with the MTR A2756G polymorphism (Table 2) in three genetic models. For G-allele vs. A-allele: OR = 1.16, 95%CI = 1.04 - 1.30, *P*_heterogeneity_ = 0.078, *P* = 0.008, *I*^2^ = 47.2 (Fig. 3). GA vs. AA: OR = 1.17, 95%CI = 1.02 - 1.33, *P*_heterogeneity_ = 0.229, *P* = 0.020, *I*^2^ = 26.1. For GG+GA vs. AA: OR = 1.18, 95%CI = 1.04 - 1.34, *P*_heterogeneity_ = 0.102, *P* = 0.010, *I*^2^ = 43.4. In subgroup analysis by race, we observed increased risk between MTR A2756G polymorphism and PCa risk in Asian population in three comparisons. For G-allele vs. A-allele: OR = 1.22, 95%CI = 1.06 - 1.40, *P*_heterogeneity_ = 0.050, *P* = 0.006, *I*^2^ = 66.6. Similar results were also indicated in a homozygote comparison (OR = 1.93, 95%CI = 1.14 – 3.26, *P*_heterogeneity_ = 0.390, *P* = 0.014, *I*^2^ = 0) and recessive genetic model (OR = 1.72, 95%CI = 1.02 – 2.89, *P*_heterogeneity_ = 0.767, *P* = 0.041, *I*^2^ = 0). In stratified analysis by tumor stage, we indicated a positive correlation between the MTR A2756G variant and advanced PCa (GG vs. GA+AA: OR = 2.31, 95%CI = 1.28 - 4.17, *P*_heterogeneity_ = 0.486, *P* = 0.006, *I*^2^ = 0), but not in localized PCa (OR = 0.81, 95%CI = 0.43 – 1.54, *P*_heterogeneity_ = 0.579, *P* = 0.252, *I*^2^ = 0) (Fig. 4). Furthermore, we observed positive correlations between MTR A2756G variant and PCa susceptibility in hospital-based studies while applying four genetic models (allelic contrast: OR = 1.19, 95%CI = 1.05 - 1.34, *P*_heterogeneity_ = 0.159, *P* = 0.007, *I*^2^ = 39.4; homozygote comparison: OR = 1.58, 95%CI = 1.03 – 2.42, *P*_heterogeneity_ = 0.444, *P* = 0.038, *I*^2^ = 0; heterozygote comparison: OR = 1.17, 95%CI = 1.01 - 1.35, *P*_heterogeneity_ = 0.175, *P* = 0.033, *I*^2^ = 36.9; dominant genetic model: OR = 1.19, 95%CI = 1.04 - 1.36, *P*_heterogeneity_ = 0.105, *P* = 0.014, *I*^2^ = 47.8).

**Table 2:** Stratified analyses of the MTR gene rs1805087 A2756G polymorphism on prostate cancer risk.

Variables	N	Cases/	G-allele vs. A-allele		GG vs. AA		GA vs. AA		GG+GA vs. AA		GG vs. GA+AA
		Controls	OR(95%CI) *P*_heter_^a^ *P* *I*^2^		OR(95%CI) *P*_heter_^a^ *P* *I*^2^		OR(95%CI) *P*_heter_^a^ *P* *I*^2^		OR(95%CI) *P*_heter_^a^ *P* *I*^2^		OR(95%CI) *P*_heter_^a^ *P* *I*^2^
Total	7	2921/3095	1.16(1.04-1.30) 0.078 0.008 47.2		1.33(0.93-1.92) 0.280 0.119 19.6		1.17(1.02-1.33) 0.229 0.020 26.1		1.18(1.04-1.34) 0.102 0.010 43.4		1.24(0.86-1.77) 0.493 0.246 0
Ethnicity											
Caucasian	3	683/639	1.03(0.85-1.24)0.897 0.792 0		0.86(0.50-1.46) 0.812 0.569 0		1.11(0.88-1.40) 0.983 0.392 0		1.07(0.86-1.35) 0.962 0.535 0		0.83(0.49-1.40) 0.810 0.478 0
Asian	3	2134/2346	1.22(1.06-1.40) 0.050 0.006 66.6		1.93(1.14-3.26) 0.390 0.014 0		1.31(0.84-2.05) 0.044 0.233 68.0		1.38(0.87-2.20) 0.024 0.174 73.2		1.72(1.02-2.89) 0.767 0.041 0
South America	1	104/110	2.77(1.05-7.29) - 0.039 -		3.42(0.35-33.49) - 0.290 -		2.57(0.77-8.02) - 0.127 -		2.74(0.93-8.07) - 0.067 -		3.24(0.33-31.63) - 0.312 -
Source of control											
Population-based	2	474/395	1.48(0.55-4.00) 0.042 0.439 75.7		0.87(0.44-1.73) 0.206 0.699 37.4		1.16(0.85-1.60) 0.182 0.344 43.9		1.13(0.84-1.53) 0.092 0.417 64.8		0.84(0.43-1.65) 0.213 0.615 35.6
Hospital-based	5	2447/2700	1.19(1.05-1.34) 0.159 0.007 39.4		1.58(1.03-2.42) 0.444 0.038 0		1.17(1.01-1.35) 0.175 0.033 36.9		1.19(1.04-1.36) 0.105 0.014 47.8		1.44(0.94-2.20) 0.734 0.093 0

^a^ *P* value of *Q*-test for heterogeneity test(*P*_heter_).

### *In-silico* analysis of MTR expression

**Figure 5:**
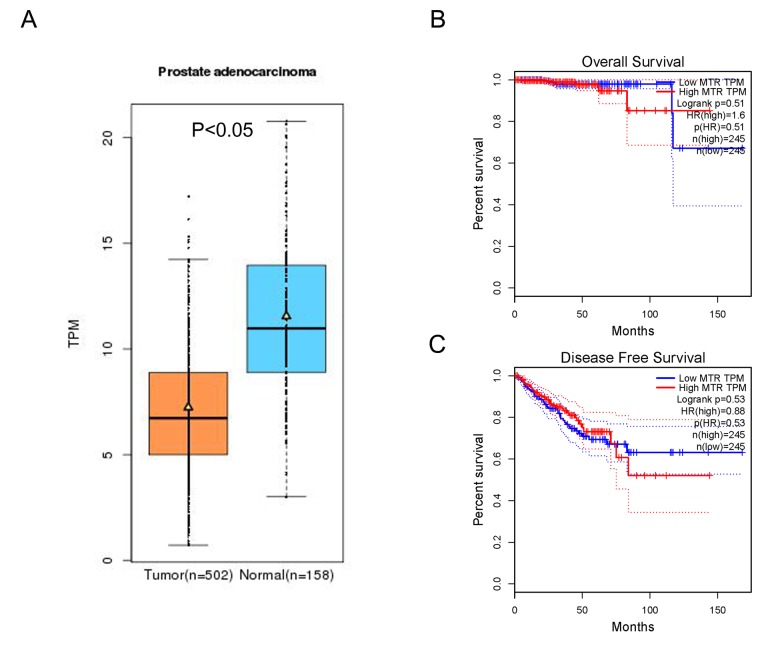
***In-silico* analysis of MTR expression.** (**A**) The relative expression of MTR in PCa tissue and paracancerous tissue (Normal) using TCGA database. TPM (Transcripts Per Kilobase Million) stands for the expression of MTR in each tissue. MTR expression in PCa tissue was lower than that in normal tissue (data from 502 PCa and 158 normal samples, *P* < 0.05). The correlation between MTR expression and overall survival time (**B**) or disease free survival time (**C**) of prostate cancer patients (*P* > 0.05).

**Figure 6:**
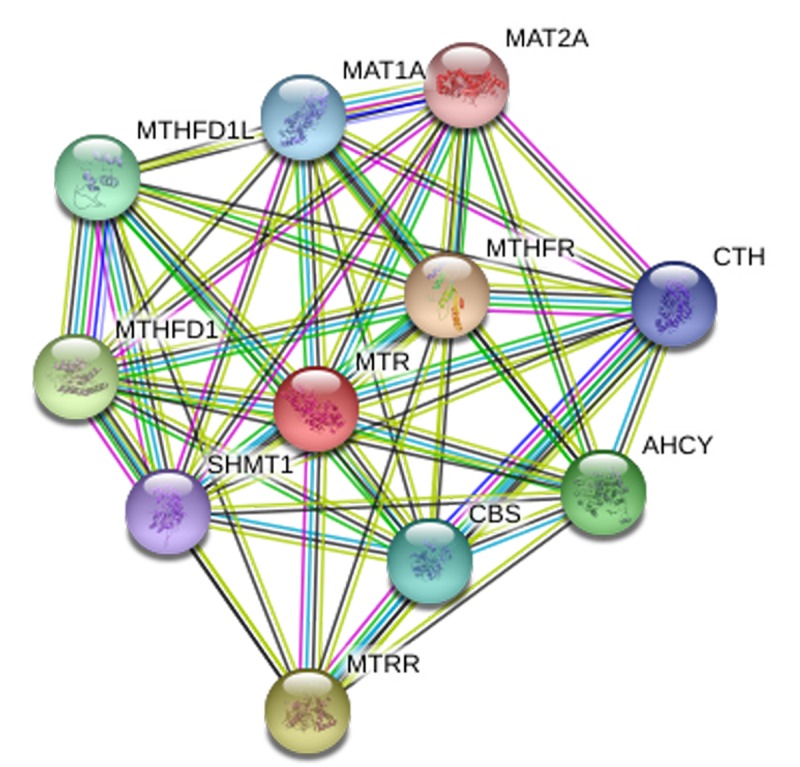
**Human MTR interactions network with other genes obtained from String server.** At least 10 genes have been indicated to correlate with MTR gene. CTH: Cystathionase; AHCY: adenosylhomocysteinase; MTRR: 5-methyltetrahydrofolate-homocysteine methyltransferase reductase; SHMT1: Serine hydroxymethyltransferase 1; MTHFD1: methylenetetrahydrofolate dehydrogenase 1; MTHFD1L: methylenetetrahydrofolate dehydrogenase 1-like;CBS: cystathionine beta-synthase; MAT1A: methionine adenosyltransferase I, alpha. MAT2A: methionine adenosyltransferase II, alpha; MTHFR: methylenetetrahydrofolate reductase.

*In-silico* analysis showed evidence that MTR expression in PCa tissue was lower than that in normal tissue (including 502 PCa and 158 normal samples, *P* < 0.05, Fig. 5 A). Furthermore, the association between MTR expression and PCa overall survival time or disease free survival time was also studied. Kaplan-Meier estimate demonstrated no significant difference for either overall survival time or disease free survival time (*P* > 0.05, Fig. 5 B). Unfortunately, we have not indicated a positive association between the MTR polymorphism and different stage (OR = 1.07, 95%CI = 0.83 - 1.40, *P* = 0.591) and grade (OR = 1.11, 95%CI = 0.85 - 1.44, *P* = 0.445) of PCa in our PCa patient study. String online server indicated that MTR gene interacts with numerous genes. The network of gene-gene interaction has been illustrated in figure 6 (Fig. 6).

### Serum expression of MTR with different genotypes in PCa patients

**Figure 7:**
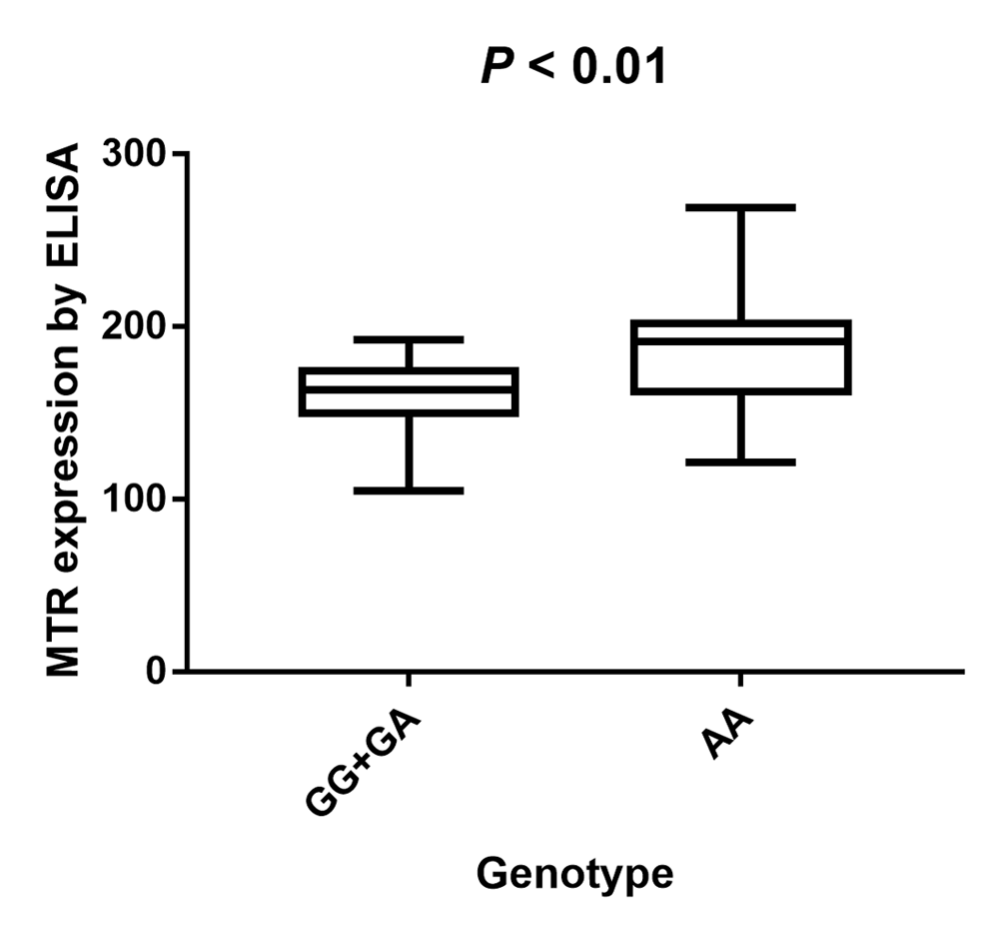
**Analysis of serum MTR levels in rs1805087 A/G genotype of PCa cases with mean values (horizontal lines, mean values).** Serum MTR levels in PCa patients carrying GG/GA genotypes were relatively lower than that carrying AA genotypes. *P* < 0.01 compared with the GG/GA and AA genotypes.

Two-hundred serum samples of PCa patients were collected in our study with different genotypes of the MTR A2756G variant. We screened the MTR gene frequency of PCa patients recruited in our hospital. The distribution for GG, GA, and AA was 27 (13.5%), 65 (32.5%), 108 (54.0%), respectively. MAF of MTR A2756G in our study participants was 0.298, which is a bit higher than the one reported for AFR population (0.284) and lower than the one reported for EAS population (0.321). *P* value for HWE in our PCa cases is 0.002. Furthermore, we investigated the serum expression of MTR by ELISA. Serum MTR levels in PCa patients with GG/GA genotypes were statistically lower than those with AA genotypes (*P* < 0.01, Fig. 7).

### Publication bias

**Figure 8:**
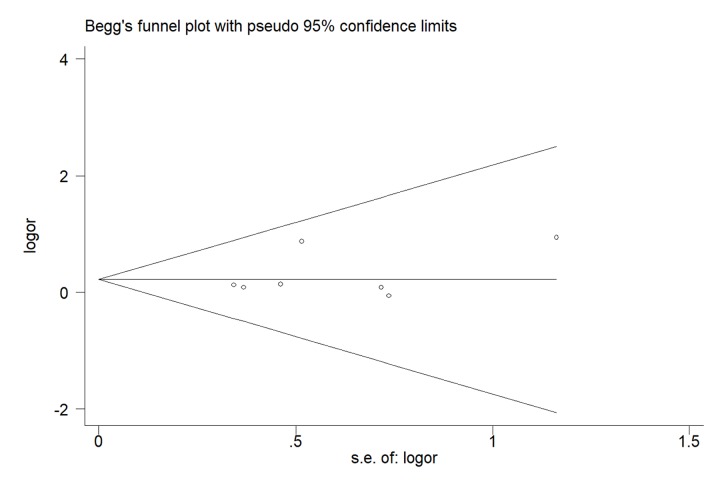
**Begg's funnel plots to examine publication bias for recessive genetic model of MTR A2756G polymorphism.** The funnel plot did not indicate any substantial asymmetry, which means no evidence of publication bias was found. t value = 0.83, *P* value = 0.445 for GG vs. GA+AA.

We used Begg’s and Egger’s test to assess publication bias of our meta-analysis. No evidence of bias was indicated in all the genetic models. For G-allele vs. A-allele: t = 1.14, *P* = 0.305; GG vs. AA: t = 0.92, *P* = 0.399; GA vs. AA: t = 1.24, *P* = 0.270; GG+GA vs. AA: t = 1.28, *P* = 0.257; GG vs. GA+AA: t = 0.83, *P* = 0.445 (Fig. 8)

## DISCUSSION

PCa remains one of the most prevalent malignancies among men globally, frequently occurring in Western countries [25,26]. Previous research has shown evidence that some risk factors, including ethnicity, toxins, lifestyle, single nucleotide polymorphism (SNP), and family history may contribute to the process and development of PCa [27–29]. Accumulating data have indicated that MTR, a folate-metabolizing gene, may be involved in the process of prostate carcinogenesis [30–32]. However, the association between MTR gene A2756G polymorphism and PCa risk remains unclear. Marchal and his group indicated that MTR 2756G allele acts as a factor of tumor aggressiveness in prostate carcinoma [33], nevertheless, some other research suggested no correlation between MTR A2756G polymorphism and PCa susceptibility [22]. Previous meta-analysis has assessed the association between MTR A2756G polymorphism and PCa in 2009 [17], however, no statistically significant correlation of this variant on the susceptibility to PCa was detected. After that, more articles have been published and provided new information to correlate MTR A2756G polymorphism and PCa susceptibility, such as Qu et al., who investigated the effect of the MTR polymorphism in the Han Chinese population and suggested that this variant was independently related to prostatic carcinogenesis by decreasing methylation potential [18]. In our present study, we selected some novel and larger case-control studies to draw a convincing conclusion. Thus, an updated meta-analysis with 2,921 PCa patients and 3,095 control subjects was undertaken to study this SNP.

In our meta-analysis, seven studies for MTR A2756G polymorphism were investigated. The overall results implicated MTR A2756G polymorphism in an increased risk of PCa. We observed that GA genotype presented 1.17-fold higher risk of PCa than the AA genotype in the overall analysis. Ethnicity is a vital biological factor; we uncovered a positive association between MTR A2756G polymorphism and PCa risk in Asian population, but not in Caucasian and other populations. Similar positive results were also observed in HB studies when stratified by source of control. To our knowledge, tumor stage is an independent prognostic factor for PCa; significant association between MTR A2756G variant and advanced PCa was indicated. In addition, we investigated the serum expression of MTR by ELISA and found that individuals who were GG/GA carriers of MTR A2756G polymorphism had a lower serum expression level than AA carriers. This result suggests that mutation from A to G at this locus (Asp to Gly substitution at codon 919 in MTR protein) may cause low expression of MTR, which means that serum concentration of MTR may be utilized as a detector in the diagnosis of PCa. To further ascertain the effect of MTR A2756G variant in PCa susceptibility, an online gene expression mini-database was utilized to evaluate the expression of MTR in PCa tissues. The Cancer Genome Atlas (TCGA) projects produced large-scale RNA sequencing data and may provide a chance for integrated expression analysis for all genes in large quantities of tumor and normal tissue specimens. It showed that MTR expression in PCa tissue was lower than that in normal tissue, which was consistent with the result from our meta-analysis. In addition, interactions of MTR with other gene might play a role in the current genetic polymorphism. As was shown in Figure 6, ten other genes may be involved in the gene-gene interaction. Further research is warrant to confirm these interactions in more details.

Although this is the first study that assesses the association between MTR A2756G polymorphism and PCa risk up to now, some limitations of the present analysis should be noted. First, all enrolled articles were the type of retrospective case-control studies, so selection bias may be existed in the present meta-analysis. Second, it is possible that gene-gene interactions influence the function of gene product and predominate in the development of cancer, which may lead to different ORs. We tried to evaluate the potential interactions, however, raw information from enrolled articles was insufficient. Some more precise researches need to be carried out if other covariates including PSA level, smoking exposure, and so on becomes available in the future case-control studies. Third, no enrolled article was carried out in African population, which should be considered in the future studies. Furthermore, age has been indicated to be associated with PCa risk, we tried to further assess the possible linkage between aging and MTR A2756G polymorphism, nevertheless, the raw data was not available either. Our PCa patients study indicated that serum MTR levels in PCa patients with AA genotypes were statistically higher than in those with GG/GA genotypes. Then, results from meta-analysis indicated a significant association between this variant and advanced PCa. Unfortunately, we have not indicated a positive association between the MTR polymorphism and different stage or grade of PCa in our case study. The possible reasons include: (a), the sample size of our experiment was not large enough; (b), there may be selection bias. In addition, MAF of MTR A2756G in our study participants was a bit higher than the one reported for AFR population (0.284) and lower than the one reported for EAS population (0.321). *P* value for HWE in our PCa cases is 0.002, which indicated that the departure of frequencies of MTR gene might exist.

Meta-analysis is considered to be an effective method for combining previously published results to draw more comprehensive conclusions. In spite of this, some other limitations should be addressed. To start with, only 7 studies based on MTR A2756G polymorphism and PCa were included, which means that the total participant count remains relatively low for comprehensive analysis. Second, only articles written in Chinese or English language were screened; hence, research published in other languages may be neglected. Third, some other risk factors, including family history, lifestyle, and environmental factors were not assessed due to insufficient data. Therefore, future research ought to collect and evaluate this information in more detail. Despite these limitations, some advantages in the present study should be acknowledged. First, heterogeneity is of great significance in meta-analysis. No obvious heterogeneity was detected in the current analysis for MTR A2756G polymorphism. Second, the quality of the selected studies completely satisfied the selection criteria and the statistical efficiency was enhanced. Third, most of the included studies, except one, are consistent with HWE [20]. Fourth, no evidence of publication bias was identified when we assessed the impact of the MTR variant in PCa risk.

In summary, our current study indicated that the MTR A2756G polymorphism was not only associated with increased PCa risk, particularly in people of Asian descent and in HB studies, but also related to PCa prognosis. Furthermore, studies with larger sample size should be continued to fully elucidate this issue.

## MATERIALS AND METHODS

### Search strategy and eligibility of relevant studies

PubMed, Embase, CNKI, and WanFang databases searches were conducted using the following keywords: ' methionine synthase ' or ' MTR ', 'prostate cancer' or 'prostate carcinoma' and 'variant' or 'polymorphism' (last search was carried out on Feb 10, 2018). References cited in the retrieved articles were also screened by a manual search. The inclusion criteria were: (a) study of association between MTR A2756G polymorphism and PCa susceptibility or prognosis; (b) available data for all genotypes (GG, GA, and AA); (c) case–control study with no duplicated design.

### Inclusion criteria, exclusion criteria, data extraction and quality assessment

The following criteria were used to screen the eligible studies: (a) evaluation of MTR A2756G polymorphism and PCa susceptibility; (b) containing available data for genotype frequencies; (c) sufficient information for evaluating ORs; (d) case-control studies. Major exclusion criteria were: (a) no available genotype frequency; (b) studies used a case-case or case-only type; (c) If authors published two or more articles using the same population, the article with most complete information was enrolled. Two investigators independently screened all the searched data from studies according to the selection criteria. Any discrepancies were fully discussed by all authors unto consensus. The following parameters from the selected studies were extracted: the first author's surname, publication year, country, race, methods of genotyping, source of control (hospital-based, HB or population-based, PB), sample size of the participants, Minor Allele Frequency (MAF) in case and control, Average age for case group and control group. Hardy-Weinberg equilibrium (HWE). In addition, the selected studies contained data on clinical stage of PCa, which were separated into two sub-groups: localized (Gleason score =< 7 or tumor stage T1-T2c) and advanced PCa (Gleason score > 7 or tumor stage T3-T4). Moreover, we used the Newcastle-Ottawa Scale (NOS) score, ranging from zero stars (worst) to nine stars (best), to assess the quality of each study. The NOS score was calculated by comparability of participants and ascertainment of exposure to risks [34]. An article with 7 stars or more can be known as a high quality one.

### Enzyme Linked immunosorbent Assay (ELISA)

We collected the blood in standard cubes without anticoagulant, then utilized serum separator tube (SST) and allow samples to clot for two hours at room temperature or overnight at 4°C before centrifugation for 15 minutes at 1000 ×g. Removed serum and assay immediately or aliquot and stored samples at -20°C or -80°C. Repeated freeze-thaw cycles should be avoided. Serum expression of MTR levels were measured by ELISA kit (CUSABIO Co. ltd.). Micro plate reader capable of measuring absorbance at 450 nm, with the correction wavelength set at 540 nm or 570 nm. The absorbance is related to the standard curve.

### *In-silico* analysis of MTR expression

The online gene expression mini database was utilized to evaluate the expression of MTR in prostate tissues (http://gemini.cancer-pku.cn/) [35]. This database contains RNA expression profiles of 502 clinically confirmed PCa samples and 158 normal parts, which were isolated from the corresponding tissues. String online server was utilized to study the network of gene-gene interaction for MTR gene (http://string-db.org/).

### Statistical analysis

We utilized odds ratios (ORs) with 95% confidence intervals (CIs) to investigate the strength of association between MTR A2756G polymorphism and PCa susceptibility. *Z*-test was used to determine the statistical significance of ORs. Chi-square-based *Q*-test was utilized to evaluate the heterogeneity assumption. A random effects model (DerSimonian-Laird method) was used when the *P*-value for the *Q*-test was less than 0.05 [36], which demonstrated a lack of heterogeneity among the studies. Otherwise, a fixed effects model (Mantel-Haenszel method) was applied [37]. *I*^2^ was also utilized to measure the heterogeneity. If *I*^2^ > 50%, this would suggest statistically significant heterogeneity among the studies. For the MTR A2756G genetic variant, the association with PCa risk was evaluated according to the models as follows: allelic comparison (G-allele vs. A-allele), homozygote comparison (GG vs. AA), heterozygote comparison (GA vs. AA), dominant model (GG+GA vs. AA) and recessive model (GG vs. GA+AA). Begg's [38] and Egger's [39] tests were chosen to calculate the funnel plot asymmetry and publication bias. *P* values less than 0.05 were considered statistically significant. We calculated the departure of frequencies of MTR gene variant from expectation under HWE by utilizing the Pearson chi-square test. Stata software (version 11.0, College Station, TX) was selected to investigate all statistical tests.

### Genotyping methods

Genotyping of MTR A2756G variant was performed utilizing various techniques in different studies, including quantitative real-time PCR, restriction fragment length polymorphism PCR (PCR-RFLP), and allele-specific Taqman probes and primers as mentioned by Ulvik [40]. In our study, MTR A2756G polymorphism was evaluated using a TaqMan assay as conducted by Li et al [41].

### Study population

Overall, 200 newly diagnosed PCa patients from February 2013 to July 2017 in the third Affiliated Hospital of Nantong University and Affiliated Changzhou No.2 People's Hospital of Nanjing Medical University were involved in our study (Table 3). The age range of the recruited PCa patients was from 52 to 85 years old. They were diagnosed with this cancer by transurethral resection of prostate (TURP) or needle biopsy within the preceding one year. Laparoscopic radical prostatectomy (LRP) was carried out on all patients and the pathological examination was performed. Every study participant should provide 2 milliliter peripheral blood sample. This study was approved by the Institution Review Board of the third Affiliated Hospital of Nantong University and Affiliated Changzhou No.2 People's Hospital of Nanjing Medical University. The written informed consent of each patient was obtained before all samples were collected.

**Table 3:** Clinicopathological and demographic characteristics of PCa patients included in this study.

Features	PCa patients
n	200
Age, n(%)	
<60	101(50.5)
≥60	99(49.5)
Sex, n(%)	
Male	102(51)
Female	98(49)
Smoking, n (%)	
Ever	89(44.5)
Never	111(55.5)
Alcohol drinking, n (%)	
Ever	121(60.5)
Never	79(39.5)
PSA, n (%)	
4-10	123(61.5)
10-20	62(31)
>20	15(7.5)
Gleason score(%)	
<7	91(45.4)
=7	62(31)
>7	47(23.5)
TNM stage(%)	
≤T2c	141(70.5)
=T3a	38(19)
≥T3b	21(10.5)
Recurrent(%)	
Yes	17(8.5)
No	183(91.5)
